# On the Feasibility of Interoperable Schemes in Hand Biometrics

**DOI:** 10.3390/s120201352

**Published:** 2012-02-01

**Authors:** Aythami Morales, Ester González, Miguel A. Ferrer

**Affiliations:** Instituto Universitario para el Desarrollo Tecnológico y la Innovación en Comunicaciones (IDeTIC), Universidad de Las Palmas de Gran Canaria, Campus de Tafira s/n, E-35017 Las Palmas de Gran Canaria, Spain; E-Mails: esterglezs@gmail.com (E.G.); mferrer@dsc.ulpgc.es (M.A.F.)

**Keywords:** biometric identification, hand based biometrics, hand geometry, palm texture, interoperability

## Abstract

Personal recognition through hand-based biometrics has attracted the interest of many researchers in the last twenty years. A significant number of proposals based on different procedures and acquisition devices have been published in the literature. However, comparisons between devices and their interoperability have not been thoroughly studied. This paper tries to fill this gap by proposing procedures to improve the interoperability among different hand biometric schemes. The experiments were conducted on a database made up of 8,320 hand images acquired from six different hand biometric schemes, including a flat scanner, webcams at different wavelengths, high quality cameras, and contactless devices. Acquisitions on both sides of the hand were included. Our experiment includes four feature extraction methods which determine the best performance among the different scenarios for two of the most popular hand biometrics: hand shape and palm print. We propose smoothing techniques at the image and feature levels to reduce interdevice variability. Results suggest that comparative hand shape offers better performance in terms of interoperability than palm prints, but palm prints can be more effective when using similar sensors.

## 1. Introduction

Our society has always placed great emphasis on maintaining the privacy of confidential information. Traditionally, a user could be identified through something known only by the user, such as a password, or something owned exclusively, for instance: a card. The main inconvenience of these methods lies in the ease of appropriating the user’s identity.

Biometric techniques help overcome these security issues. Specifically, biometric systems take advantage of physical or behavior features during the identification process. When a biometric trait is assumed, it is necessary to consider four fundamental characteristics: universality, uniqueness, invariance and quantification. Quality-cost relation and convenience have to be taken into account when the biometric technology is transferred to the industry. Robustness evaluation is also needed with the aim of minimizing vulnerability.

The main biometric systems measuring physical features are based on finger print, face, hand-shape, palm print and iris recognition. Examples of behavior biometric systems are hand-writing, signature and voice. In this paper we focus our attention on two of the most popular hand biometrics: hand shape and palm prints. The reliability of hand shape and palm print biometrics is high enough to be used in realistic and low cost environments. Furthermore, these systems allow researchers to use different hand traits available with just one shot and even to combine them without any additional hardware cost. In addition, hand-based biometric systems present a high level of acceptability from their users.

So far the scientific community has presented a large variety of different biometric systems based on hand shape and palm prints. Their proposals can be classified according to different biometric considerations. One such consideration concerns the acquisition device used, such as scanners [1–5], CCD cameras [6–8] and webcams [9–11]. Another classification can be done according to the hand side: palm [1,2,5,8,12–16] or dorsum [6,7,17]. The illumination spectrum used varies from the visible [3,6–8,13,18–20] to the near infrared [21,22] and multispectral imaging [23,24]. The variety on the proposals is wide [25] but to our knowledge there are few studies regarding the relationships between the different schemes or approaches [26].

In 2006 the NSTCs Subcommittee on Biometrics of the U.S Government developed The National Biometrics Challenge [27] to identify key challenges in advancing the development of biometrics. The report concludes that to fully meet large-scale identity governance requirements, the use of biometric technology must be more robust, scalable and interoperable.

**Figure 1.**
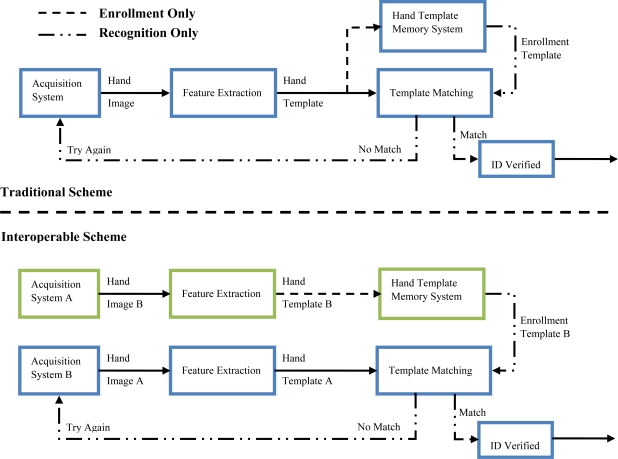
Hand biometric recognition approaches for traditional and interoperable schemes.

Interoperability is one of the aspects of biometry that has been scarcely studied. This property provides a measure of the performance when you enroll a user with a biometric device A and verifies his identity with a biometric device B (see Figure 1). Working with interoperable procedures reduces technological dependences between users, models and systems and allows companies to upgrade their biometric devices without the cost of repeated enrolment of all the users.

### 1.1. Our Work

In this paper we present a study of interoperable procedures for hand shape and palm print biometrics. The first contribution is a database made up of 8,320 hand images using six different acquisition systems including a scanner, CCD cameras and CMOS-webcams. In terms of biometric approaches the experiments include dorsum and palm with contact or contactless imaging. Every set of images from each user in the database was acquired under the same conditions trying to ascertain fair terms to benchmark the interoperability between schemes. A second contribution of the study is the resultant comparison on the performance of four state-of-the-art feature extraction methods over multiple scenarios using traditional and interoperable schemes. The third contribution is the proposal of a smooth operator to reduce interdevice variability and improve interoperability between schemes.

The paper is organized as follows: in Section 2 we analyze the state of the art in hand shape and palm print biometrics and interoperability studies proposed using other biometrics. Then, in Section 3, we describe the different biometric schemes used to build the database while Section 4 reviews proposed feature extraction methods for the hand shape. Palm print features are introduced in Section 5. In Section 6 we present the interoperable database and evaluation methodology in order to illustrate our results. Conclusions are given in Section 7.

## 2. State of the Art

### 2.1. Hand Shape Biometric

The very first publications on hand shape biometry date back to 1999 [6]. This system is based on images of the hand dorsum acquired with CCD sensors and uses pegs to guide hand placement. Systems using pegs are not suitable for the natural posture of the hand and the simplicity of the system, and soon peg-free systems were introduced [28]. In this case, scanners were used as acquisition systems to acquire an image of the palm of the hand.

A first set of features used by hand-shape biometric systems was geometric measurements. Standard measures include: the length and width of the fingers [5,7,17,21], hand contour [6], area of the palm or the fingers [19] or the thickness of the hand [29]. Geometric measurements based on 3-D surface curvature features have also been proposed [15–17]. The number of geometric features of a traditional geometric template in the literature ranges between 13 and 40 [25].

A second method to parameterize hand shape is to model the hand silhouette by a curve where the curve coefficients represent the hand shape features. These methods are based either on Principal Component Analysis (PCA) or Independent Component Analysis (ICA) [1,30]. Another way to parameterize the hand silhouette uses alignment procedures [20].

Lastly, the introduction of contactless systems brought about to improvements in hygienic measures and acceptability levels from users. Zheng *et al*. [19] used invariants to projection measures with limited results. Later, Morales *et al.* [21] proposes a contactless biometric system based on the geometry of the hand in the infrared band which allows for hand segmentation in not-controlled backgrounds. Recent publications explore the tridimensional information of the fingers [15,16].

The results for the performance of biometric system based on hand shape features reveal values ranging from 5% to 0% of the EER that depends on the methodology and database employed, (see Table 1). The absence of a common benchmark to compare different approaches is an important fault on hand shape biometrics, although public hand shape databases are available [31,32].

### 2.2. Palm Print Biometrics

Palm print studies depend on the resolution of the images. If high resolution images are taken (400 dpi or more), singular point minutia can be extracted as main features. When low resolution images are used (in the range of 150 dpi or less) the study has to focus on features such as principle lines, wrinkles and texture. The applications for each type of image are also diverse. For instance, high resolution images are suitable for forensic applications while low resolution images are used in commercial applications and access control. Most of the palm texture or palm print studies found in the literature focus on low resolution images.

Two different types of low resolution palm print acquisition systems are generally used: those based on CCD cameras [3,18,22,24,33–36] and those based on digital scanners [2,3,4,37]. This second group is usually slower and bigger than the first one. The major problem with a scanner-based device is the distortion due to the pressure by the hand on the scanner screen. Palm print devices can also be classified depending on whether they use pegs [3] or are peg-free [37] which improves user acceptance. Another classification is by contact [3,4,14,18,33–37,] or contactless [9–11,24,38–40] and has to handle the distortion caused by projection, blurriness caused by hand movement and changes in illumination (see Table 2).

The most popular procedures to obtain palm print features can be divided into global appearance features such as CompCode, OLOF or Wavelet [9,18,33,35] and local information features such as SIFT or SURF [3,10,34]. The use of public databases such as the PolyU Palm print database [41] or the IITD database [42] is common and allows for comparisons between proposals under fair conditions.

### 2.3. Interoperability

The above systems evaluate training and testing of the hand biometric trait with the same device. Our research on interoperability revealed only a few published contributions about hand shape or palm print biometric systems interoperability [26], and as such this area is now extended by the present paper. Regarding other biometric traits, several papers have been published about finger print device interoperability [14,43–48] or sensor interoperability for signature verification [49].

In [41] the researcher explores the interoperability between three different finger print sensors, one with sweeping thermal and two with optical technology. The results show that the performance drops dramatically for interoperable schemes and multi-instance schemes were proposed to improve the results. Similar results were reported in [14].

The relationships among person, sensor and feature for finger print recognition were discussed in [48]. The authors deal with the idea that the problems of sensor interoperability originate from two main factors: the performance gap between different sensors and the drop of performance caused by coordinating different sensors.

The influence of quality in interoperable schemes for signature verification was discussed in [49]. The authors state that performance is primarily affected when using more reliable sensors for training and therefore it is crucial to have enrolment models that are generated with high quality data.

All the previous works related to interoperability between biometric systems confirm an observed drop in performance when using interoperable schemes. There is still much work to do to reach a future in which the use of interoperable schemes allows for the employment of biometric data that has been acquired with different sensors and approaches.

## 3. Biometric Acquisition Devices

During all these years, biometric systems based on hand shape and palm print have been widely studied. Therefore, the study of hand shape and palm print interoperability should consider as many existing devices as possible ranging systems based on scanner, CCD camera, webcams, contactless systems, systems based on palm and the dorsum of the hand, *etc.* Taking the above mentioned considerations into account, we have used six different hand-based biometrics approaches which are briefly described in this section.

The images from different system present different resolution and quality. The characteristics of the images are heterogeneous and although hand shape biometrics can achieve promising results using low resolution images (40 dpi) in order to ensure competitive performance for palm print biometrics a higher resolution is needed (at least 60 dpi). In our proposals we assume this heterogeneity and we include it as a factor to overcome in order to achieve interoperable schemes.

First we describe the systems that acquire images of the hand dorsum. Then we continue with the systems that acquire images of the palm side of the hand.

### 3.1. Systems Acquiring Images of the Hand Dorsum

**Figure 2.**
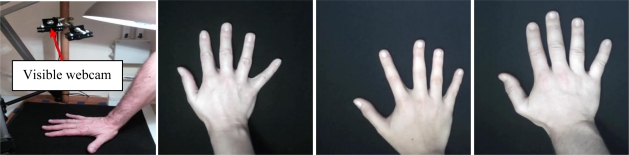
Hand dorsum visible acquisition system (figure on the left) and examples of visible hand images from three different users (three figures on the right).

**Figure 3.**
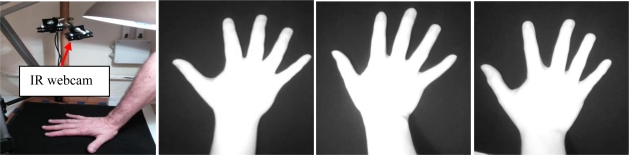
Hand dorsum infrared acquisition system (figure on the left) and examples of acquired IR hand images from different users (three figures on the right).

Systems acquiring hand dorsum images are illustrated in Figures 2 and 3. As seen in the illustration, the user puts the hand on a plate. The cameras and the illumination are above the hand. Two cameras are used and operate in the visible and infrared bands.

- **System 1 (Visible webcam):** Biometric system based on a visible webcam. As seen in Figure 2, this webcam acquires the complete image of the dorsum in the visible band (400–750 nm). The result is a 640 × 480 pixel image.
- **System 2 (Webcam 850 nm):** Biometric system based on an infrared webcam. This biometric system acquires the hand dorsum image by means of a webcam which has been modified in order to take images in the 850 nm band of the infrared region. The webcam modification consists of removing the visible filter and inserting an infrared filter instead. The result, shown in Figure 3, is a 640× 480 pixel image.

### 3.2. Systems Acquiring the Hand by the Palm Side

In this case the user puts the hand on a flat glass. The acquisition system and illumination are below the glass. Two acquisition systems are used: a scanner and a CCD camera.

**Figure 4.**
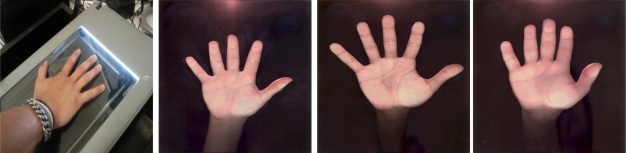
Scanner acquisition (figure on the left) and acquired hand images from three different users (three figures on the right).

**Figure 5.**
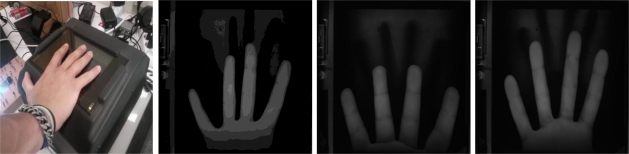
PRM acquisition device (figure on the left) and acquired IR hand images from three different users (three figures on the right).

- **System 3 (Scanner):** Biometric system based on a scanner. This is a traditional approach. The user places his hand upon the scanner with the fingers outstretched. Moreover, the fingers cannot touch the boundaries of the scanner. The scanner works at 60 dpi with 256 levels of gray resulting in 701 × 509 images. Figure 4 illustrates the procedure and the images acquired in the visible band.
- **System 4 (PRM):** a biometric system based on a CCD camera. In this case we used the PRM233c Big Eye device. This device was developed by the Hungarian Recognition company and was designed with the aim of acquiring passport images in visible, infrared and ultraviolet bands. We only used this device for hand shape biometric identification purposes in the infrared band. Health considerations prevented the use of ultraviolet images. The illumination is based on white and infrared LEDs. Device space limitations reduced the number of fingers in the images (thumb is discarded). Images are captured with 2,048 × 1,595 pixel size, as seen in Figure 5.

### 3.3. Contactless Systems Acquiring the Hand by the Palm Side

**Figure 6.**
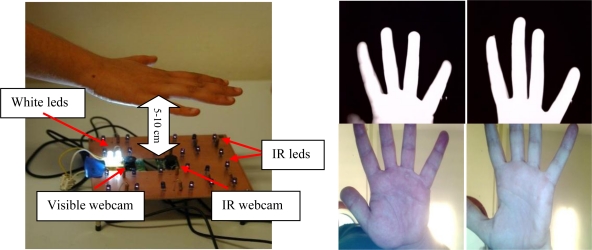
Contactless acquisition system at a short distance (figure on the left) and acquired IR and visible hand images from two different users (four figures on the right in upper level IR images; in down level visible images).

These systems are developed to verify users through hand geometry in the infrared band. The Infrared band is used to improve segmentation of the hand in open conditions. For that purpose, the parameters of the infrared webcam are adjusted to a small exposition time, low brightness, high contrast and gain. As a result the hand image is saturated as can be seen in the last row of Figure 6, where precise segmentation of the hand from the unconstrained background is easily done.

The illumination consists of 16 infrared LEDs in the 850 nm band. These LEDs are located around the webcam. The location was designed to illuminate the hand evenly. The infrared webcam used acquire images of 1,600 × 1,200 pixels. According to the distance from the hand to the webcam, we have two different biometric systems:

- **System 5 (Contactless 5–10 cm):** Contactless images acquired at a short distance (in the 5–10 cm range). Figure 6 shows the assembly, acquisition procedure and acquired images sample.

The user is asked to keep his/her hand over the webcam, in the range of 5–10 cm. Thus, the infrared webcam acquires only an image of the fingers, very similar to the PRM233C images, while the visible image is focused on the palm. This scheme produces uncorrelated IR and visible images but the short distance and the high resolution (1,600 × 1,200) increase-the data quality.

- **System 6 (Contactless 20–30 cm):** Contactless images acquired at a medium distance (in the 20–30 cm range).

**Figure 7.**
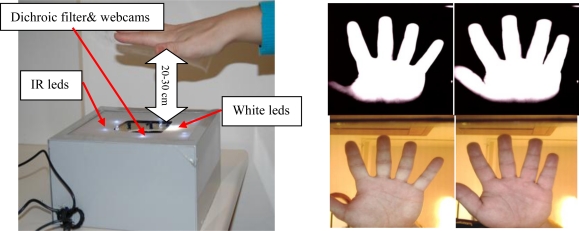
Contactless acquisition system at a medium distance (figure on the left) and acquired IR and visible hand images from two different users (4 figures on the right in upper level IR images; in down level visible images).

With this biometric system, an infrared and visible image of the complete hand is taken. The hand of the user has to be around 20–30 cm from the webcams, see Figure 7.

The use of a dichroic filter (Cold Mirror) allows a correlated hand image in both IR and visible domains to be obtained, although it requires a greater distance in comparison with system 5. This distance degrades the image quality.

## 4. Hand Shape Features Extraction Methods

We explore the usability of two traditional hand shape feature extraction methods based on finger geometry measures and global appearance features. While geometric features involve local information about widths, lengths, angles or sizes the global appearance features consider the complete scene image.

### 4.1. Finger Geometry Feature Extraction Method

Geometric features extracted from the hand-shape are obtained from a traditional finger geometry feature extraction method. The geometric features are obtained by measuring 100 widths of each finger starting with the 15th point and ending with the 85th point of the finger length. As several of the hand shape biometric devices proposed in this paper just acquire the finger area, it is not possible to include palm measures because they cannot be taken in all the devices. Due to the acquisition devices setup and illumination, a reliable hand contour can be obtained through binarization of the grayscale images with its Otsu’s threshold [50].

To work out the tips and valleys between the fingers we convert the Cartesian coordinates of the contour to polar coordinates (radius and angle) considering the center of the image first row (wrist size) as the coordinate’s origin. The peaks in the radius coordinate locate the provisional position of the finger tips and the minimum of the radius indicate the valleys between fingers. Let r_c_(i) and *ϕ_c_*(*i*), 1 ≤ *i* ≤ *L* be the radius and angle of the *i^th^* hand contour pixel. The index $i_{p}^{f}$ of the *i^th^* radius peaks are obtained as:

(1) $$i_{p}^{f}\;\in \;\;\mathrm{peak}\;\;\mathrm{if}\;r_{c}\left(i_{p}^{f}\right)=\max\left\{r_{c}(i)|i_{p}^{f}-100\leq i\leq i_{p}^{f}+100\right\}$$

with $101<i_{p}^{1}<i_{p}^{2}<\cdots i_{p}^{5}<L-100$. If the number of radius peaks obtained is greater than 5, we suppose than the hand detector has been fault and go back to the hand detection module waiting for a hand. As the hand is expected, $i_{p}^{1}$ corresponds to the little finger tip. The index $i_{v}^{j}$ of the *j^th^* valley is worked out as:

(2) $$i_{v}^{f}\;\in \mathrm{valley}\;\mathrm{if}\;r_{c}(i_{v}^{f})=\min\{r_{c}(i)|i_{p}^{f}\leq i\leq i_{p}^{f+1}\}$$

The exterior base of the index and little fingers are obtained as the nearest pixel of the exterior contour to the valley between the index and middle fingers and the valley between the index and little fingers, respectively, *i.e.*,:

(3) $$i_{\mathrm{index}}=\underset{i_{p}^{4}\leq i\leq i_{p}^{5}}{\mathrm{argmin}}\{d(\langle x_{c}(i),\;y_{c}(i)\rangle ,\;\langle x_{c}(i_{v}^{3}),\;y_{c}(i_{v}^{3})\rangle )\}$$

(4) $$i_{\mathrm{little}}=\underset{1\leq i\leq i_{p}^{1}}{\mathrm{argmin}}\{d(\langle x_{c}(i),\;y_{c}(i)\rangle ,\;\langle x_{c}(i_{v}^{1}),\;y_{c}(i_{v}^{1})\rangle )\}$$

being *d*(·, ·) the Euclidean distance. We will call $i_{v}^{0}=i_{\mathrm{little}}$, and $i_{v}^{4}=i_{\mathrm{index}}$.

**Figure 9.**
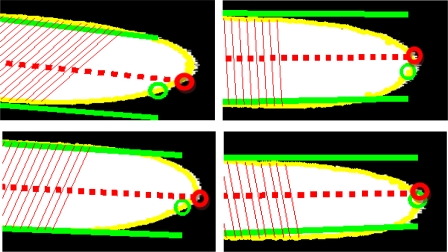
Initial tip localization and finger sides (in green); finger axis and accurate tip localization (in red).

The position of the tip of the finger is finely adjusted as follows:

**Step 1.** Four equally spaced points are selected, starting with the 35th point and ending with the 80th point of each finger side. The 35% piece is selected to avoid the presence of rings, and the 80% piece is selected to avoid the tip curvature of the finger tip. For the right side of the finger, the four points are calculated as $i_{\mathrm{rfs}}^{f}\;(k)=\left(i_{p}^{f}-i_{v}^{f-1}\right)*C(k)+i_{v}^{f-1}$, being *C*(*k*) = {0.35, 0.50, 0.65, 0.80}, and for the left finger side the points are calculated as $i_{\mathrm{lfs}}^{f}\;(k)=\left(i_{v}^{f+1}-i_{p}^{f}\right)*C(k)+i_{p}^{f}$.
**Step 2.** The lines that minimize the square error with the selected point of each finger side are calculated. For the right side, the line is defined as $y=m_{r}^{f}\cdot x+b_{r}^{f}$, being $b_{r}^{f}$ and $m_{r}^{f}$ calculated as:
  (5) $$\left(\begin{array}{c} b_{r}^{f}\\ m_{r}^{f} \end{array}\right)=\mathrm{pinv}\;\left(\begin{array}{cc} 1 & x(i_{\mathrm{rfs}}^{f}(1))\\ 1 & x(i_{\mathrm{rfs}}^{f}(2))\\ 1 & x(i_{\mathrm{rfs}}^{f}(3))\\ 1 & x(i_{\mathrm{rfs}}^{f}(4)) \end{array}\right)\cdot \left(\begin{array}{c} y(i_{\mathrm{rfs}}^{f}(1))\\ y(i_{\mathrm{rfs}}^{f}(2))\\ y(i_{\mathrm{rfs}}^{f}(3))\\ y(i_{\mathrm{rfs}}^{f}(4)) \end{array}\right)$$
  being *pinv* the pseudoinverse. For the left side, the line $y=m_{l}^{f}\cdot x+b_{l}^{f}$ is obtained as above using $i_{\mathrm{lfs}}^{f}(k),\;k=1,\ldots ,4$, Figure 9.
**Step 3.** The average of the two lines is considered the finger axis and calculated as $y=m_{a}^{f}\cdot x+b_{a}^{f}$ being $m_{a}^{f}=(m_{r}^{f}+m_{l}^{f})/2$ and $b_{a}^{f}=(b_{r}^{f}+b_{l}^{f})/2$, see Figure 9.
**Step 4.** The tip of the finger is the point where the finger axis and the finger contour intersect, Figure 9.

(6) $$i_{p}^{f}=\underset{i_{v}^{f-1}\leq i\leq i_{v}^{f+1}}{\mathrm{argmin}}\left\{d_{y=m_{a}^{f}\cdot x+b_{a}^{f}}\;\left(x_{c}(i),\;y_{c}(i)\right)\right\}$$

where $d_{y=m_{a}^{f}\cdot x+b_{a}^{f}}\;(\cdot )$ is the Euclidean distance with the line $y=m_{a}^{f}\cdot x+b_{a}^{f}$.

The geometric features are obtained by measuring the widths of each finger as follows: the center base of each finger $\langle x_{\mathrm{fb}}^{f},\;y_{\mathrm{fb}}^{f}\rangle$ is defined as the point where the finger axis $y=m_{a}^{f}\cdot x+b_{a}^{f}$ intersects the finger base line:

(7) $$y=\frac{y(i_{v}^{f})-y(i_{v}^{f-1})}{x(i_{v}^{f})-x(i_{v}^{f-1})}\;\;\left(x-x(i_{v}^{f-1})\right)+y(i_{v}^{f-1})$$

We select 12 equally spaced points between $\langle x_{\mathrm{fb}}^{f},\;y_{\mathrm{fb}}^{f}\rangle$ and $\langle x_{c}(i_{p}^{f}),\;y_{c}(i_{p}^{f})\rangle$ as follows:

(8) $$x_{s}^{f}\;(k)=\left(x_{c}\;(i_{p}^{f})-x_{\mathrm{fb}}^{f}\right)*C(k)+x_{\mathrm{fb}}^{f}$$

(9) $$y_{s}^{f}(k)=m_{a}^{f}\cdot x_{s}^{f}(k)+b_{a}^{f}$$

with *C*(*k*) = linespace(0.15,0.85, *n*). The perpendicular line to the finger axis is obtained in this point as:

(10) $$y=\frac{-1}{m_{a}^{f}}\;\left(x-x_{s}^{f}(k)\right)+y_{s}^{f}(k)=m_{\mathrm{pa}}^{f}\cdot x+b_{\mathrm{pa}}^{f}$$

**Figure 10.**
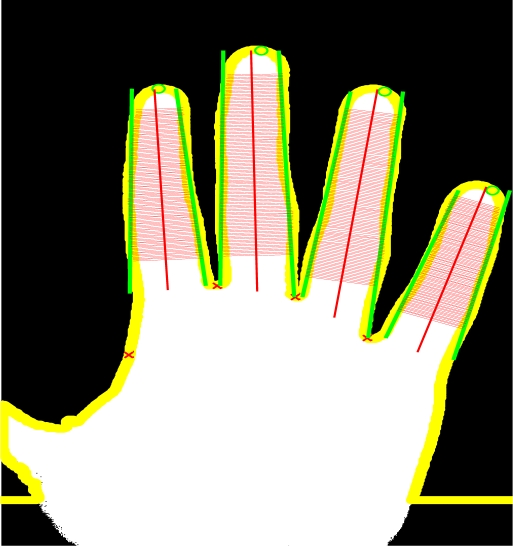
Finger widths measured for the geometrical template.

The nearest contour points to this line are:

(11) $$i_{\mathrm{cr}}^{f}\;(k)=\underset{i_{v}^{f-1}\leq i\leq i_{p}^{f}}{\mathrm{argmin}}\left\{d_{y=m_{\mathrm{pa}}^{f}\cdot x+b_{\mathrm{pa}}^{f}}\;\left(x_{c}(i),\;y_{c}(i)\right)\right\}$$

(12) $$i_{\mathrm{cl}}^{f}\;(k)=\underset{i_{p}^{f}\leq i\leq i_{v}^{f}}{\mathrm{argmin}}\left\{d_{y=m_{\mathrm{pa}}^{f}\cdot x+b_{\mathrm{pa}}^{f}}\left(x_{c}(i),\;y_{c}(i)\right)\right\}$$

being the width at this point $d_{w}^{f}\;(k)=d\left(\langle x_{c}\left(i_{\mathrm{cr}}^{f}\;(k)\right),\;y_{c}\left(i_{\mathrm{cr}}^{f}\;(k)\right)\rangle ,\;\langle x_{c}\left(i_{\mathrm{cl}}^{f}\;(k)\right),\;y_{c}\left(i_{\mathrm{cl}}^{f}\;(k)\right)\rangle \right)$. The geometric features are obtained by measuring 100 widths of each finger, starting with the 15th point and ending with the 85th point of the finger length. An example can be seen in Figure 10.

The width measures of the four fingers are concatenated resulting in a vector of 400 components $d_{w}^{f}\;(k)$, 1 ≤≤4, 1≤ *k* ≤ 100.

**Smoothing procedure to improve the inter-device variability:** obviously, not all the devices are able to obtain the same hand contour. The contour obtained with the hand dorsum is not equal to the silhouette obtained by the palm side. Therefore, besides the well-known translation, rotation and temporal variability, an interoperable algorithm has to cope with device variability. The within person inter device contour variability should be reduced while minimizing the reduction of the inter person variability. Given that averaging or smoothing are procedures that reduce the variance a bias of a measure, our proposal is to accomplish the inter device robustness task by using a smooth operator at two levels: the first one smoothing the hand contour and the second with a low pass filter applied to the feature vector.

- **First level (contour level):** Let ${\left\{x_{i},\;y_{i}\right\}}_{i=1}^{L}$ the *L* 8-connected pixels that define the hand contour. The smoothed contour is obtained with a moving averaging filter of order equal to 11. Therefore the smoothed contour ${\left\{xs_{i},\;ys_{i}\right\}}_{i=1}^{L}$ is obtained as:
  (13) $${\mathrm{xs}}_{i}=\frac{1}{11}\sum_{j=i-5}^{j=i+5}x_{j}$$
  (14) $${\mathrm{ys}}_{i}=\frac{1}{11}\sum_{j=i-5}^{j=i+5}y_{j}$$
- **Second level (feature level):** the projection distortion can be reduced by first setting the mean value of the smoothed feature vector tuned to zero and dividing by its maximum value and then subtracting its average. The Discrete Cosine Transform (DCT) is applied to the smoothed normalized feature vector and the new geometrical hand template is obtained by selecting from the 2nd to the 50th coefficients of the DCT transform which corresponds to the lower frequencies and is equivalent to a new smoothing.

As a verifier we used a Least Squares Support Vector Machine (LS-SVM). SVMs have been introduced within the context of statistical learning theory and structural risk minimization. Least Squares Support Vector Machines (LS-SVM) are reformulations to standard SVMs which lead to solving linear KKT systems. Robustness, sparseness, and weightings can be imposed on LS-SVMs where needed and a Bayesian framework with three levels of inference is then applied [51].

The meta-parameters of the LS-SVM model are the width of the Gaussian kernels σ and the regularization factor *γ*. The regularization factor is taken as *γ* = 20 and is identical for all the LS-SVM models used here. The Gaussian width σ parameter is optimized as follows: the training sequence is randomly partitioned into two equal subsets *P_i_*, 1 ≤ *i* ≤ 2. The LS-SVM is trained *L* = 30 times with the first subset *P*_1_ and Gaussian width equal to *L* logarithmically equally spaced values between 10^1^ and 10^4^*σ_l_*, 1 ≤ *l* ≤ *L*. Each one of the *L* LS-SVM models is tested with the second subset *P*_2_ obtaining *L* Equal Error Rate *EER_l_*, 1 ≤ *l* ≤ *L* measures. The positive samples are trained with target output +1 and the negative samples with target value −1. The Gaussian width *σ* of the signature model is obtained as *σ* = *σ_j_*, where *j* = *argmin*_1 ≤ *l* ≤ *L*_{*EER_l_*}. Finally, the user hand model is obtained by training the LS-SVM with the complete training sequence.

### 4.2. Global Appearance Hand Shape Feature Extraction Method

The global appearance feature extraction method proposed in [1] is adapted for interoperable schemes. The hand shape obtained by the different systems proposed only warranted the complete image of four of the five fingers of the hand. The high pose variability of the thumb finger decreases the overall performance [25] and this degradation is greater in contactless schemes. Therefore we propose a global appearance feature extraction method based on the complete image of the four fingers: index, middle, ring and little.

**Figure 11.**
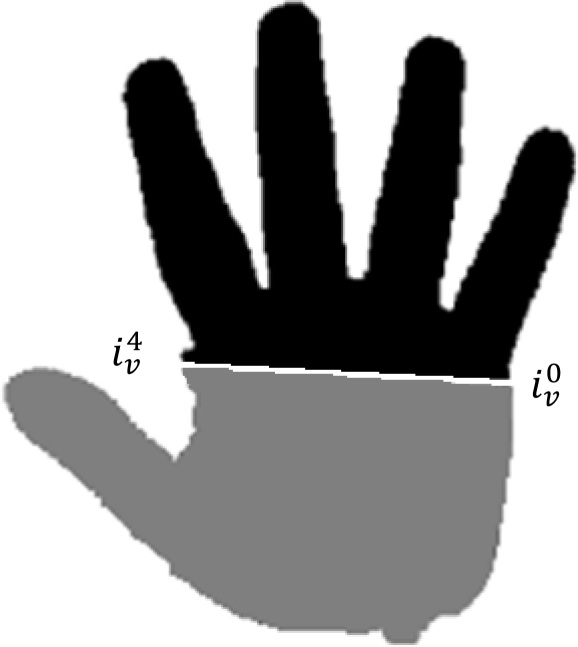
Black regions are the regions of interest when employing the hand shape global appearance features while discarded regions are in gray.

**Figure 12.**
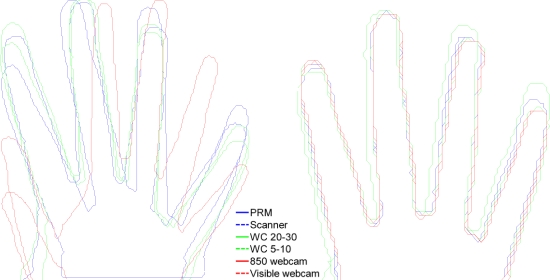
Superposed hand silhouettes registration images before finger normalization (left) and after finger normalization (right).

The feature extraction methods based on global appearance features are strongly dependent on the normalization of the hand image. The registration of hand images involves the normalization of the global rotation, translation and the re-orientation of the fingers individually along standardized directions. The method proposed can be divided into four steps:

- **First step:** translation to the centroid of the hand so that it coincides with the center of the image.
- **Second step:** rotation toward the direction of the larger eigenvector. The eigenvector corresponds to the largest eigenvalue of the covariance matrix of the object coordinates and the angle can be obtained as:
  (15) $$\emptyset =0,5\arctan\;\left(\frac{2\mu_{1,1}}{\mu_{2,0}-\mu_{0,2}}\right)$$
  where *μ*_1,1_, *μ*_2,0_ and *μ*_0,2_ are the second-order centered moments of the binary hand pixel distances from their centroid.
- **Third step:** discarding of thumb and palm regions using the line obtained by the points $i_{v}^{0}$ and $i_{v}^{4}$, Figure 11.
- **Fourth step:** the necessity of finger re-orientation is illustrated in Figure 12. The figure shows hand shapes of the same person acquired with the six proposed systems after global hand registration (without finger normalization).

The finger normalization algorithm is made up of the following three phases [1]: (a) extracting fingers: using the location of tips and valleys presented in Section 4.1; (b) finger pivots: fingers rotate around the joint between the proximal phalanx and the corresponding metacarpal bone; (c) hand pivotal axis: each finger is rotated by the angle, for the index, middle, ring, little, and the position of the goal orientation of that finger. The finger rotations are effected by multiplying the position vector of the finger pixels by the rotation matrix around their pivot:

(16) $$R_{\theta }=\left[\begin{array}{cc} \cos\theta & -\sin\theta \\ \sin\theta & \cos\theta \end{array}\right]$$

The standard angles are defined as {120,100,80,60} for the index, middle, ring and little finger respectively. After normalizing the finger orientations, the hand is once again translated and rotated so that its centroid, defined as the mean of the four pivot points, is moved to a fixed reference point in the image plane. The complete hand image is rotated so that its pivot line aligns with a fixed chosen orientation and resized to 200 × 200 pixels image. The result of the complete hand normalization procedure can be seen in Figure 12.

As seen the finger normalization corrects the pose distortion and reduces the inter-device variability but this correction is not fully accomplished in contactless imaging due to the high projective distortion present in an unconstrained acquisition.

Once normalized, the feature extraction method employed is based on ICA_2_. The data vectors for the ICA_2_ decomposition are the lexicographically ordered hand image pixels. The dimension of these vectors is 200 × 200 = 40,000 features. The distance *d_ICA_* between two features vectors *f_i_* and *f_j_* is computed in terms of cosine of the angle between them as:

(17) $$d_{\mathrm{ICA}}=\text{arg}\;\max_{i}\left\{\frac{f_{i}\cdot f_{j}}{\left\|f_{i}\right\|\;\left\|f_{j}\right\|}\right\}$$

## 5. Palm print Feature Extraction Methods

This section presents palm print features as an alternative to the hand shape features previously described and as a way to combine different biometric traits of the hand in interoperable schemes. This paper explores the interoperability of the palm print biometric trait using two of the most promising palm print features approaches based on either global appearance features (OLOF) or local information features (SIFT).

### 5.1. Palmprint ROI Extraction

**Figure 13.**
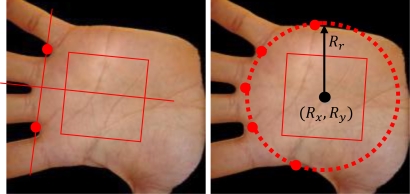
Palmprint ROI extraction methods, traditional [33] method (left) and proposed method (right).

The distortions associated with inter-device variability and the projective distortions associated with contactless schemes introduce errors on the location and size of the Region of Interest (ROI). In this study we propose a ROI extraction method for interoperable palm print recognition based on the addition of three extra points to the traditional approach based on two points [33]. The center and the size of the ROI are located minimizing the quadratic error of the circumference obtained by the Cartesian coordinates of the points $c_{i}={\left\{x_{c}(i_{v}^{f}),\;y_{c}(i_{v}^{f})\right\}}_{f=0}^{4}$, being $c_{i}={\left\{x_{v}^{f},\;y_{v}^{f}\right\}}_{f=1}^{3}$ the Cartesian coordinates of the valleys, $c_{0}(x_{v}^{0},\;y_{v}^{0})$ and $c_{4}(x_{v}^{4},\;y_{v}^{4})$ defined as the points of the silhouette at $i_{v}^{0}-100$ and $i_{v}^{4}+100$ respectively. The circumference which minimizes the mean quadratic error is calculated by the pseudovector:

(18) $$\left(\begin{array}{c} R_{x}\\ R_{y}\\ R_{r} \end{array}\right)=\mathrm{pinv}\;\left(\begin{array}{ccc} 2x_{v}^{0} & 2y_{v}^{0} & 1\\ 2x_{v}^{1} & 2y_{v}^{1} & 1\\ 2x_{v}^{2} & 2y_{v}^{2} & 1\\ 2x_{v}^{3} & 2y_{v}^{3} & 1\\ 2x_{v}^{4} & 2y_{v}^{4} & 1 \end{array}\right)\cdot \left(\begin{array}{c} x_{v}^{0}\;x_{v}^{0}+y_{v}^{0}\;y_{v}^{0}\\ x_{v}^{1}\;x_{v}^{1}+y_{v}^{1}\;y_{v}^{1}\\ x_{v}^{2}\;x_{v}^{2}+y_{v}^{2}\;y_{v}^{2}\\ x_{v}^{3}\;x_{v}^{3}+y_{v}^{3}\;y_{v}^{3}\\ x_{v}^{4}\;x_{v}^{4}+y_{v}^{4}\;y_{v}^{4} \end{array}\right)$$

where *R_x_* and *R_y_* are the coordinates of the center of the circumference and *R_r_* is the ratio (Figure 13).

The image inside the circumference image is rotated along the angle of the finger axis $y=m_{a}^{3}\cdot x+b_{a}^{3}$ to obtain rotation invariability and the ROI is defined by the square centered at the coordinates *R_x_* and *R_y_* with side length equal to 0.75 × *R_r_*. With this method the location and size of the ROI depends of the five points which reduce the inter-device variance (e.g., scale variability due to different acquisition schemes). The image is processed by contrast limited adaptive histogram equalization [52] to improve contrast between palm lines. The palm print image is finally resized by bilinear interpolation to 150 × 150 pixels in order to apply the same feature extraction algorithms to all the schemes.

### 5.1. Global Appearance Features Extraction Method

The Orthogonal Line Ordinal Features (OLOF) method was originally introduced in [18] and was investigated for application in palm print feature extraction. The comparison of OLOF method with several other competing methods [3] suggests its superiority in several palm print biometric devices. We worked out the OLOF features as defined in [18]. This method is based on 2D Gaussian filter to obtain the weighted average intensity of a line-like region. Its expression is as follows:

(19) $$f(x,\;y,\;\theta )=\text{exp}\;\left[-{\left(\frac{x\cos\theta +y\sin\theta }{\delta_{x}}\right)}^{2}-{\left(\frac{-x\sin\theta +y\cos\theta }{\delta_{y}}\right)}^{2}\right]$$

where *θ* denotes the orientation of 2D Gaussian filter, *δ_x_* denotes the filter’s horizontal scale and *δ_y_* denotes the filter’s vertical scale parameter. There are no significant differences on the results in the range *δ_x_*, *δ_y_* ∈ [0.5 − 10].

To obtain the orthogonal filter, two Gaussian filters are used as follows:

(20) $$\mathrm{OF}(\theta )=f(x,\;y,\;\theta )-f\left(x,\;y,\;\theta +\frac{\pi }{2}\right)$$

**Figure 14.**
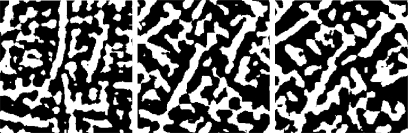
Palm print OLOF features for OF(0), OF(π/6) and OF(π/3).

Each palm image is filtered using three ordinal filters *OF*(0), *OF*(*π*/6) and *OF*(*π*/3) to obtain three binary masks based on a zero binarization threshold. In order to ensure the robustness against brightness, the discrete filters *OF*(*θ*), are turned to have zero average. Finally, the three images are reduced to 50 × 50 pixel. An example of these images can be seen in Figure 14.

In order to verify that a query texture *Q* belongs to the identity with image texture (training) *P* we used a normalized Hamming measure which can be described as:

(21) $$D=1-\frac{\sum_{i=1}^{2n+1}\sum_{j=1}^{2n+1}P(i,j)⊗Q(i,j)}{{\left(2n+1\right)}^{2}}$$

where the Boolean operator ⊗ is equal to zero if and only if the two pixels *P*(*i*, *j*) and *Q*(*i*, *j*) are equal. Note that D is between 0 and 1 (best matching). Due to imperfect preprocessing, we need to translate vertically and horizontally one of the features to a range of 4 to 4 and match again. The maximum D value obtained is considered to be the final matching score.

### 5.1. Local Information Features Extraction Method

**Figure 15.**
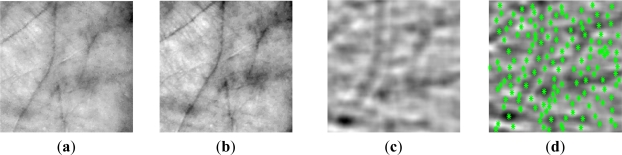
Original palmprint image (**a**); contrast limited adaptive histogram equalized palmprint (**b**); Gabor filtered palmprint (**c**) and keypoint location on Gabor filtered image (**d**).

The Scale Invariant Feature Transform (SIFT) was originally proposed in [53]. The features extracted are invariant to image scaling, rotation, and partially invariant to illumination changes and projective distortion. With a goal to reduce the within person interdevice variability when searching the SIFT keypoints, we preprocess the gray scale palm print image by filtering it with a 2D even Gabor filter. This procedure, called Modified SIFT (MSIFT), has already been used in [54] in order to make the SIFT key point extortion more robust to contactless distortions. The SIFT algorithm is based on detecting keypoints with similar properties that are present in the reference and questioned image. The MSIFT consists of 6 steps:

- **Preprocessing:** we assume that training and the questioned hand have a similar orientation inside the image (it is achieved during the segmentation stage). The real 2D Gabor filter used to process the palm print image is defined by:
  (22) $$G(x,\;y,\;\theta ,\;u,\;\sigma )=\frac{1}{2\pi \sigma^{2}}\exp\;\left\{-\frac{x^{2}+y^{2}}{2\sigma^{2}}\right\}\;\exp\{2\pi i(\mathrm{ux}\cos\theta +\mathrm{uy}\sin\theta )\}$$
  where *u* is the frequency of the sinusoidal wave, θ defines the orientation selectivity of the function, and σ is the standard deviation of the Gaussian envelope. We used a Gabor filter setting with *θ* = 0, *σ* = 2.0 and u = 0.1. Greater robustness against brightness variation is assured by turning the discrete Gabor filter to average zero:
  (23) $$\tilde{G}=(x,\;y,\;\theta ,\;u,\;\sigma )=G(x,\;y,\;\theta ,\;u,\;\sigma )-\frac{\sum_{i=-n}^{n}\sum_{j=-n}^{n}G(i,\;j,\;\theta ,\;u,\;\sigma )}{{\left(2n+1\right)}^{2}}$$
- **Scale-space extrema detection:** It is applied over all scales and image locations. It is based on the difference-of-Gaussian function to identify potential interest points that are invariant to scale and orientation. The input data is transformed to the space *L*(*x*, *y*, *σ*) as follows:
  (24) $$L(x,\;y,\;\sigma )=G(x,\;y,\;\sigma )*I^{\prime }(x,\;y)$$
  where * corresponds to the operator convolution, *I*′(*x*, *y*) is the preprocessed input image and *G*(*x*, *y*, *σ*) is a Gaussian function with bandwidth *σ*. The difference-of-Gaussian function is defined as:
  (25) D(x, y, σ)=(G(x, y, kσ)−G(x, y, σ))*I(x, y)=L(x, y, kσ)−L(x, y, σ)
- **Keypoint localization:** A detailed model is fit to determine the location and scale of each candidate location. The interpolation is done using the quadratic Taylor expansion of the Difference-of-Gaussian scale-space function *D*(*x*, *y*, *σ*) with the candidate keypoint as the origin. This Taylor expansion is given by:
  (26) $$D(x)=D+\frac{\partial D^{T}}{\partial x}+\frac{1}{2}\;x^{T}\;\frac{\partial^{2}D^{T}}{\partial x^{2}}x$$
  where the maximum and minimum of *D* and its derivatives are evaluated at the candidate keypoint and *x* = (*x*, *y*, *σ*) is the offset from this point [Figure 15(d)].
- **Orientation assignment:** In our experiments we used 16 orientations for each keypoint location based on local image gradient directions. For an image sample *L*(*x*, *y*) at scale *σ*, the gradient magnitude *m*(*x*, *y*), and orientation, *θ*(*x*, *y*), are processed using pixel differences:
  (27) $$m(x,\;y)=\sqrt{{\left(L(x+1,\;y)-L(x-1,\;y)\right)}^{2}+{\left(L(x,\;y+1)-L(x,\;y-1)\right)}^{2}}$$
  (28) $$\theta (x,\;y)=\tan^{-1}\left(\frac{L(x,\;y+1)-L(x,\;y-1)}{L(x+1,\;y)-L(x-1,\;y)}\right)$$
- **Keypoint descriptor:** Around each keypoint, the local gradients are measured at the selected scale to obtain a descriptor vector ${\left\{d_{i}\right\}}_{i=1}^{M}$ with *M* keypoints. Once the keypoints are extracted, the query image is matched and compared with each of the features extracted with the corresponding images in the registration database (from the training feature sets). The verifier evaluates the number of matches between the queried and the training images. Let ${\left\{d_{i}^{t}\right\}}_{i=1}^{M}$ and ${\left\{d_{j}^{q}\right\}}_{j=1}^{L}$ be the set of training and questioned keypoint descriptors respectively. The distance between keypoint descriptors is computed from the following:
  (29) $$D_{d}(i,\;j)={\left\|d_{i}^{t}-d_{j}^{q}\right\|}^{2}$$
  where ‖·‖ is the Euclidean norm. We define a match between a training $d_{i}^{t}$ and a questioned $d_{j}^{q}$ keypoint when:
  (30) $$1.5\;D_{d}(i,\;j)<\min{\left\{D_{d}(i,\;n)\right\}}_{n=1,n\neq j}^{L}$$
  with *n* ≠ *j*. The threshold is estimated heuristically during the training stage and it is not particularly sensitive to values in the range of 1.2 to 1.7.
- **Matches Validation:** The validation of matching scores for the authentication decisions is common in several other biometric feature extraction approaches. In this paper we propose a validation based on coordinates distance between keypoints to improve the SIFT performance on the contactless palm print biometrics. The hypothesis is that the coordinates from two keypoints matched must be similar if we correct the average displacement from all the matches. Let $c_{i}^{t}={\left\{x_{i}^{t},\;y_{i}^{t}\right\}}_{i=1}^{M}$ and $c_{j}^{q}={\left\{x_{j}^{q},y_{j}^{q}\right\}}_{j=1}^{L}$ be the set of training and questioned keypoint coordinates respectively. The distance between coordinates is calculated from the following:
  (31) $$D_{c}(i,\;j)={\left\|c_{i}^{t}-c_{j}^{q}\right\|}^{2}$$
  where ‖·‖ is the Euclidean norm. We define a match between a training $c_{i}^{t}$ and a questioned $c_{j}^{q}$ keypoint when:
  (32) $$D_{c}(i,\;j)<\frac{1.5}{M}{\sum_{i=1}^{M}\left\|c_{i}^{t}-c_{j}^{q}\right\|}^{2}$$

Due to high pose variance in contactless imaging we used a 1.5 weighting factor to accommodate small alignment errors between palms. The number of matches between the questioned and the training set is the similarity score.

## 6. Experiments

### 6.1. Experimentation Methodology

**Figure 16.**
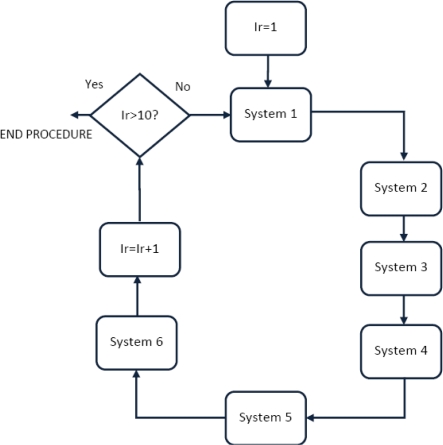
Procedure followed by the user to acquire all their samples for the interoperable database.

We acquired 10 images with each of the six schemes (note that system 5 and system 6 provide 20 images each) from 104 users in one session: for a total of 104 × 10 × 8 = 8,320 images. The decision for a unique session ensured an acquisition in the same environment conditions for all the schemes. Pose variability was maintained as follows: first we force the user to go through all the different biometric systems in an alternately way, meaning going through system 1, then system 2 up to system 6 and then returning to system 1 and repeating the process 10 times, (see Figure 16).

The experiments are tested with a close-set paradigm. Specifically from 10 images per user, we take four images for the training set, and we use the remainder in the verification phase. The experiments were designed to measure the interoperability between hand shape biometric devices and palmprint biometric devices. The interoperability experiments are carried out as follows. Interoperability entails enrolling a user with a biometric system A, and then being tested with a biometric system B. Concisely, the identifier is trained with four hands acquired from system A and tested with all the systems according to the interoperable scheme proposed on Figure 1.

### 6.2. Individual Results

**Figure 17.**
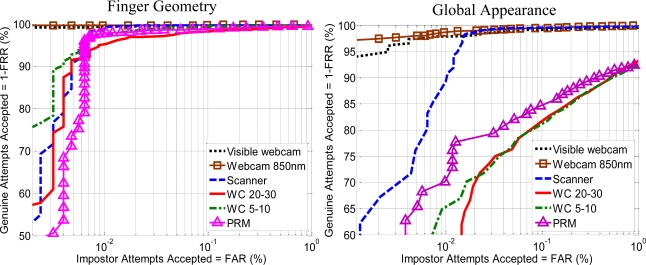
ROC curves for hand shape features extraction methods, geometric (left) and global appearance (right).

The first experiments evaluate the performance of each features approach (hand geometry, hand shape, palm print with OLOF and MSIFT) with every biometric device (non-interoperable). The value in this first experiment is to compare different hand biometric approaches with the same users and acquisition conditions. Figure 17 shows the ROC curves obtained with the geometric and shape hand features with the six devices.

**Figure 18.**
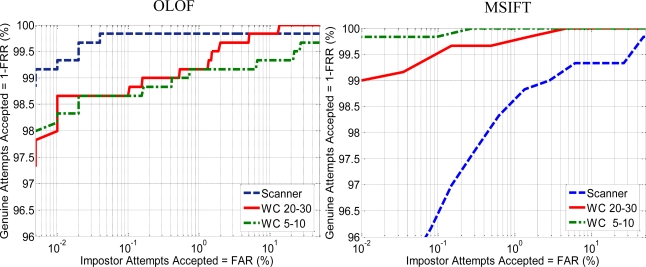
ROC curves for palm print features extraction methods, OLOF (left) and MSIFT (right).

The results for both feature extraction methods suggest a better performance by dorsum approaches. In the case of contactless schemes the results obtained are similar in both 5–10 cm and 20–30 cm scenarios. The scanner outperforms the PRM but offers lower performance than the dorsum acquisition with webcam. Figure 18 shows the results that compares the two proposed palm print feature extraction methods. The results for OLOF suggest that contact schemes offer a better performance in comparison with contactless schemes. Also the OLOF’s performance of contactless schemes with 5–10 cm or 20–30 cm distances is quite similar.

The results of MSIFT show important differences with previous OLOF results. In this case contactless scheme at 5–10 cm distance clearly outperforms scanner and contactless at 20–30 cm. schemes. The improvement is related with the resolution of the palm print images (Table 3). Although palmprint images are re-sized to 150 × 150 pixel before applying the feature extraction method, the resolution of the original image remains as an important factor on the quality of the biometric data.

While global appearance features such as OLOF can achieve promising results using low resolution images, the local information approaches such as SIFT need more resolution to achieve better performance.

### 6.3. Interoperability Results for Hand Shape Biometrics

The results in terms of Equal Error Rate (EER) are shown in Tables 4 and 5; Table 4 displays the results for finger geometry features without smoothing while Table 5 shows the results for global appearance features. The diagonal from both Table 4 and Table 5 displays how each scheme performs itself using a traditional biometric recognition approach. Although Tables 4 and 5 are near symmetric, it is obvious that it is not the same to be trained with system A and tested with B than *vice versa*.

As seen from the above results in, Tables 4 and 5, by applying traditional feature extraction methods in multiple scenarios it is possible to achieve competitive performance in individual schemes but not in interoperable schemes. In interoperable scenarios it is necessary to apply additional techniques to reduce inter-scheme variability. Table 6 shows the results of hand geometry features after applying the proposed smoothing techniques.

Analyzing the results, it is easy to conclude that smoothing improves interoperability performance. An exception occurs in the case of systems that acquire the hand image by the dorsum. We believe that this exception is related to the poor resolution and accuracy of the contours. The smoothing operation decreases the inter-device variability but also reduces the inter-user variability that defines the False Rejection Rate. This reduction is not crucial with high resolution images but it is a problem with the low resolution images of the dorsum approaches proposed.

As seen in the diagonal of Table 6, the results obtained by each individual system are closer than those in Table 4. It can be interpreted that smoothing reduces the inter-device variability of the parameters which help understand the generalization ability of the smoothing operator. Table 6 shows that the EER of the hand biometric systems based on the hand dorsum side goes from 0.25% (averaging cells {1,1} and {2,2} of Table 6 being {a,b} the result of training with system A and testing with system B), to 3.97% (averaging cells {1,2} and {2,1} of Table 6) when we change the test scheme. In the case of hand palm with contact, changing the test device worsened the EER from 0.38% (averaging cells {3,3} and {6,6} of Table 6) to 2.46% (averaging cells {6,3} and {3,6} of Table 6.) In the case of contactless, the EER goes from 1.03% (averaging cells {4,4} and {5,5} of Table 6) to 1.95% (averaging cells {4,5} and {5,4} of Table 6) when changing the scheme.

Measuring interoperability between touch devices acquiring the hand image by dorsum or palm side, the EER goes to 7,88% (averaging cells {1,3}, {1,6}, {2,3}, {2,6}, {3,1}, {3,2}, {6,1} and {6,2} of Table 6.). Obviously it worsened to 19,66% (averaging cells {1,4}, {1,5}, {2,4}, {2,5}, {4,1}, {4,2}, {5,1} and {5,2} of Table 6.) when compared to touch and contactless systems. A summary of the averaged interoperability results obtained dividing the experiments by sensors and approaches is seen in Table 7.

We can also deduce from Tables 5, 6 and 7 that the worst interoperability results are obtained when we mix contact with contactless devices and palm with dorsum devices. However, the interoperability between either contact or contactless or palm and dorsum devices need further research and improvements.

### 6.4. Interoperability of Multi-Instance Biometric Schemes Based on Finger Geometry

In this section we analyze an interoperable scenario in which the training data include two schemes, A and B, and the verification data belongs to a third scheme C. In this scenario the hand model is trained with dorsum and palm schemes or contact and contactless schemes. Although several methods have been proposed for fusion of biometric data, this study uses a simple score fusion based on a SUM rule such as:

(33) $$S_{\mathrm{ABC}}=S_{A-C}+S_{B-C}$$

where *S_ABC_* is the combined score, *S_A–C_* is the score obtained by training with system A and verifying with system C and *S_B–C_* is the score obtained by training with system B and verifying with system C.

Table 8 shows the interoperable results when using multi-instance biometric schemes for the most representative combinations. Table 8 also includes a comparison with the EERs result obtained with single-instance schemes from Table 6. The multi-instance approach improves the poor interoperable rates between contact and contactless schemes or palm and dorsum schemes. The combination at the classification score level using a traditional sum rule shows how it is possible to achieve competitive interoperable schemes with EER under the 2%.

### 6.5. Interoperability Results for Palmprint Biometrics

Interoperability results for palmprint approaches are shown in Table 9. An analysis of the interoperability between touch and contactless devices reveals that the error goes to 15.88% and 36.38% for OLOF and MSIFT, respectively (averaging cells {3,4}, {3,5}, {4,3} and {5,3} of Table 9). Once again a boundary emerges when mixing contact and contactless devices. The interoperable rates between contactless schemes go to 15.4% and 1.35% for OLOF and MSIFT, respectively (averaging cells {4,5} and {5,4} of Table 9).

From the experiment it can be deduced that global appearance features such as OLOF show more stable interoperability performance but local information approaches such as MSIFT can be more effective when using similar sensors.

The distortion due to the pressure of the hand on the scanner screen decreases the interoperable performance with contactless devices using both OLOF and MSIFT approaches. The local information feature approach MSIFT yields the best interoperability rate with 0.95%. In this case the interoperability is not symmetric which means that training with a contactless 5–10 cm system and testing with a contactless 20–30 system produces better performances than *vice versa*. It can be deduced that the greater quality and resolution of the images obtained with the contactless 5–10 system improve the training data and produces a more robust training set.

### 6.6. Interoperability Results Combining Hand Shape and Palmprint Biometrics

Combining scores obtained from different procedures is a standard practice to improve the performance of a biometric scheme. In this section we explore how the combination of hand traits improves the performance of biometric devices in interoperable scenarios. Concisely, we will combine the hand geometry and MSIFT feature extraction methods due to the better performance that those features showed on interoperable schemes. We assume that the matching scores from both features are widely separated. Prior to combining these scores, we normalize the data based on max/min approach [55]. It is then possible to combine them at score level fusion based on a linear score combination functions such as:

(34) $$s_{\mathrm{comb}}=ws_{\mathrm{geom}}+(1-w)s_{\mathrm{MSIFT}}$$

where *s_geom_* and *s_MSIFT_* are the scores obtained for geometric and palmprint biometrics respectively, *w* is the weighting factor and *s_comb_* is the combined score which will be used to verify the input identity.

The value of *w* is obtained as follows. Let $s_{\mathrm{geom}}^{g}(i)$ and $s_{\mathrm{MSIFT}}^{g}(i)\;1\leq i\leq N_{g}$the scores of the genuine training samples. Let $s_{\mathrm{geom}}^{f}(i)$ and $s_{\mathrm{MSIFT}}^{f}(i)\;1\leq i\leq N_{f}$ be the scores of the impostor training samples. A distance measure between the distribution of genuine and impostor scores is obtained for hand geometry as follows:

(35) $$\Delta_{v}={\left(m_{\mathrm{geom}}^{g}-m_{\mathrm{geom}}^{f}\right)}^{T}\sum \left(m_{\mathrm{geom}}^{g}-m_{\mathrm{geom}}^{f}\right)$$

where $m_{\mathrm{geom}}^{g}=\sum_{i=1}^{N_{g}}\;s_{\mathrm{geom}}^{g}(i)/N_{g}$, $m_{\mathrm{geom}}^{f}=\sum_{i=1}^{N_{f}}s_{\mathrm{geom}}^{f}(i)/N_{f}$ are the means, $\sum =(\sum_{\mathrm{geom}}^{g}+\sum_{\mathrm{geom}}^{f})/2$ with $\sum_{\mathrm{geom}}^{g}=\sum_{i=1}^{N_{g}}{\left(s_{\mathrm{geom}}^{g}(i)-m_{\mathrm{geom}}^{g}\right)}^{2}/N_{g}$ and $\sum_{\mathrm{geom}}^{f}=\sum_{i=1}^{N_{f}}{\left(s_{\mathrm{geom}}^{f}(i)-m_{\mathrm{geom}}^{f}\right)}^{2}/N_{f}$ the covariance matrixes. The distance between genuine and forgeries for *Δ_MSIFT_* is obtained in the same way. The weighting factor is obtained as: *w* = *Δ_MSIFT_* / (*Δ_geom_* + Δ*_MSIFT_*).

The database proposed only allows the combination in three of the six systems because the dorsum schemes and PRM device do not provide palm print imaging. The results are given in Table 10.

As seen, the combination clearly improves the previous results. The individual performance of all system is 0.01%. The combination improves the interoperability between scanner and contactless devices and allows promising results to be obtained for the interoperability of both contactless schemes with EERs of 0.02% and 0.32%.

An interoperable scheme which involves contact and contactless schemes raise doubts (EERs equal to 11.5%, 11.6%, 89.87% and 9.13%; see Table 10) but a multi-instance algorithm could alleviate these results. Additionally, the results for a contactless interoperable scheme suggest that when using multimodal schemes it is possible to achieve competitive interoperable rates with performances similar to traditional schemes.

### 6.7. User Convenience and Acceptance in Multiple Hand Based Biometric Schemes

The performance of biometric schemes or approaches is not the only important aspect in biometric recognition systems. Characteristics such as scalability, usability or convenience among others are important factors to ensure the correct transfer between laboratory and industry. We include the results of a survey made to all the users from the database with queries about the convenience and acceptance of the different hand biometric schemes proposed. The survey includes four questions and their answers are given:

The results of the survey about the hygienic and privacy concerns are not surprising. But the results about the contact or contactless preferences and comfort are unexpected. The preference of the scanner system instead of another more convenient system such as contactless is new. The easier acquisition and the familiarity with the scanner device are the main reason for these answers.

## 7. Conclusions

In this paper we have illustrated the impact of changing sensors and approaches on the performance of two of the most popular hand biometrics. The experiments show that interoperability is possible between systems based on a similar design, that is to say, between systems that acquire the hand dorsum or palm side, between touch or touchless systems, etc. although the performance with respect to use of the same device worsened by 3 to 10. The use of multi-instance or multi-modal schemes clearly outperforms the interoperable rates and emerges as the best way to achieve competitive interoperable performances.

Comparative hand shape shows better performance in terms of interoperability than palm print analysis. We deduce that the hand shape is not so dependent on the sensor. In this case, the pose and hand side (palm or dorsum) are important factors. Nevertheless the texture of the palm can vary depending on the sensor or the light used and for a fair comparison the experiments they should be done in an open set.

Future studies could include more stable parameters that are oriented to interoperability, in addition to other hand biometrics traits, such as knuckles [39,40]. The increase in the number of users on the database is essential in order to obtain more reliable conclusions. The effects of multisession acquisition to the interoperability it is also an interesting topic to explore.

## Figures and Tables

**Figure 8.**
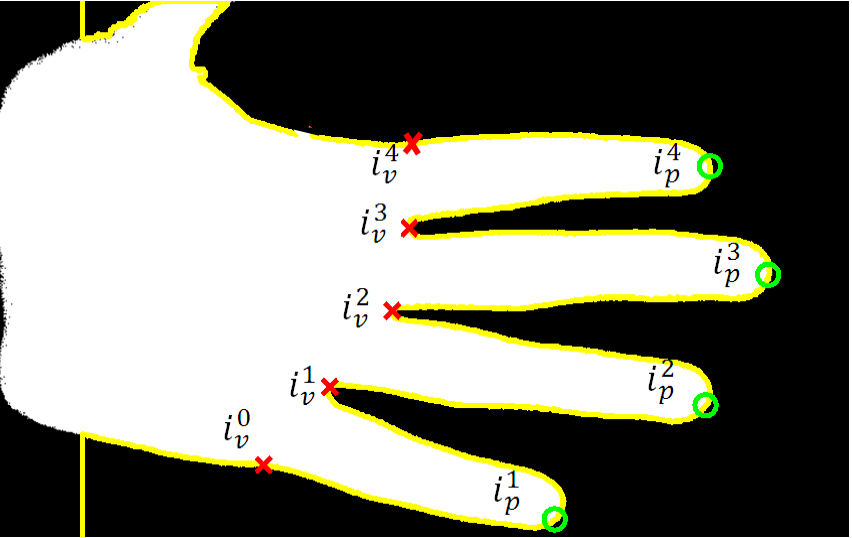
Tips, valleys and exteriors of fingers localization.

**Table 1.** Some examples of hand-shape based biometric approaches.

**Year[Ref]**	**Population**	**Methodology**	**Sensor**	**Features**	**Performance (%)**
**CONTACT SCHEMES**					
**1999 [6]**	53	Visible (Dorsum)	CCD	Hand contour coordinates	FAR = 1, FRR = 6
**1999 [7]**	20	Visible (Dorsum)	CCD	Finger lengths, widths, ratios thickness, deviation	EER = 5
**2004 [5]**	70	Visible(Palm)	Scanner	Geometric features	FAR = 1, FRR = 3
**2006 [1]**	458	Visible(Palm)	Scanner	ICA2 on binary hand image	EER = 1.3
**2008 [8]**	470	Visible (Palm)	CCD	Non-landmark based geometric measurements	FAR = 0.45, FRR = 3.4
**CONTACTLESS SCHEMES**					
**2006 [17]**	73	3D (Dorsum)	3D camera	Width and curvature of the fingers in 3D	EER = 3.6
**2007 [19]**	23	Visible (Palm)	CCD	Projective-invariant features	EER = 0
**2008 [21]**	30	IR (Palm)	Webcam	Finger widths	EER = 4.2
**2009 [15]**	177	3D-2D (Palm)	3D camera	Fusion of 3D finger curvature and 2D finger measures	EER = 2.6
**2011 [16]**	177	3D-2D (Palm)	3D camera	2D and 3D features combined at score level	EER = 0.22

**Table 2.** Some examples of palmprint based biometrics approaches.

**Year[Ref]**	**Population**	**Methodology**	**Sensor**	**Features**	**Performance (%)**
**CONTACT SCHEMES**					
**2003 [33]**	386	Visible (Palm)	CCD	Gabor filtered masks	EER = 0.6
**2005 [18]**	100	Visible (Palm)	CCD	Orthogonal Line Ordinal Feature	EER = 0.22
**2005 [4]**	75	Visible (Palm)	Scanner	PCA, FDA and ICA features	FAR = 1.35, FRR = 1.49
**2009 [3]**	100	Visible (Palm)	Scanner	Speeded Up Robust Features	FAR = 0.02, FRR = 0.01
**2009 [3]**	200	Visible (Palm)	CCD	Speeded Up Robust Features	FAR = 0, FRR = 0
**2008 [34]**	386	Visible (Palm)	CCD	SAX and SIFT	EER = 0.37
**2008 [22]**	120	IR (Palm)	CCD	LaplacianPalm based on PCA	FAR = 0.1, FRR = 0.3
**CONTACTLESS SCHEMES**					
**2007 [9]**	49	Visible (Palm)	Webcam	Circular Gabor filtering	EER = 1.2
**2007 [39]**	40	Visible (Palm)	PDA	Sum-Difference Ordinal Filtering	EER = 0.92
**2008 [10]**	320	Visible (Palm)	Webcam	Local Binary Patterns	EER = 1.52
**2008 [24]**	165	Multi (Palm)	CCD	Orthogonal Line Ordinal Features [ref]	EER = 0.5
**2010 [40]**	114	Visible (Palm)	3D camera	Fusion of 2D and 3D palm surface imaging	EER = 0.71
**2010 [11]**	100	Visible (Palm)	Webcam	Sobel filtering over multiple frame acquisitions	EER = 3.62

**Table 3.** Mean resolution (pixels) of hand images and palm print ROI of the proposed devices.

**Device**	**Image Size**	**ROI Size**

Scanner	701 × 509	115 × 115
WC 5–10	1,600 × 1,200	600 × 600
WC 20–30	1,600 × 1,200	400 × 400

**Table 4.** Interoperability matrix for Finger-Geometry approaches in terms of EER (%).

			**Training System**	**Test System**					
	**Biometric Device**	**Resolution**		**1**	**2**	**3**	**4**	**5**	**6**
Dorsum	Visible webcam	640 × 480	1	1.19	1.49	14.18	26.93	26.24	20.40
	Webcam 850 nm	640 × 480	2	2.04	1.08	13.50	23.65	26.13	17.15
Palm	Scanner	701 × 509	3	11.24	9.71	0.13	14.60	18.31	3.36
	Contactless 20–30 cm	1,600 × 1,200	4	30.80	35.55	19.89	4.91	11.26	23.29
	Contactless 5–10 cm	1,600 × 1,200	5	37.59	40.85	26.29	11.18	5.60	34.99
	PRM	2,048 × 1,595	6	20.76	17.78	4.54	16.74	23.11	1.94

**Table 5.** Interoperability matrix for Hand-Shape Global Appearance approaches in terms of EER (%).

			**Training System**	**Test System**					
	**Biometric Device**	**Resolution**							
				**1**	**2**	**3**	**4**	**5**	**6**
Dorsum	Visible webcam	640 × 480	1	0.45	7.19	11.46	29.98	31.08	46.78
	Webcam 850 nm	640 × 480	2	7.53	0.26	20.38	33.53	34.89	46.57
Palm	Scanner	701 × 509	3	10.78	18.71	0.26	20.80	20.72	35.08
	Contactless 20–30 cm	1,600 × 1,200	4	24.19	29.43	16.95	3.87	3.59	39.33
	Contactless 5–10 cm	1,600 × 1,200	5	24.74	28.30	17.69	3.92	3.77	39.33
	PRM	2,048 × 1,595	6	43.08	45.0	36.54	37.91	37.13	3.90

**Table 6.** Interoperability matrix for Finger-Geometry approaches with smoothing in terms of EER (%).

			**Training System**	**Test System**					
	**Biometric Device**	**Resolution**							
				**1**	**2**	**3**	**4**	**5**	**6**
Dorsum	Visible webcam	640 × 480	1	0.25	3.46	10.90	18.00	15.86	14.07
	Webcam 850 nm	640 × 480	2	4.48	0.26	7.12	14.66	14.86	9.65
Palm	Scanner	701 × 509	3	8.20	5.30	0.25	8.44	9.01	2.08
	Contactless 20–30 cm	1,600 × 1,200	4	22.53	23.24	11.03	0.83	1.92	13.61
	Contactless 5–10 cm	1,600 × 1,200	5	22.46	25.73	14.77	1.98	1.24	18.64
	PRM	2,048 × 1,595	6	13.16	9.73	2.84	8.78	10.59	0.51

**Table 7.** Average Interoperability between different hand shape approaches in terms of EER (%).

**Systems involved**	**Interoperability between devices with the next properties**	**Global Appearance**	**Geometry unsmoothed**	**Geometry Smoothed**
1, 2	Contact, hand Dorsum image	7.36	1.76	3.97
3, 6	Contact, Palm side images	35.81	3.95	2.46
4, 5	Contactless and Palm	3.75	11.22	1.95
3, 4, 5, 6	Contact and contactless by palm side	28.73	21.41	11.85
1, 2, 3	Contact with Palm and dorsum sides	15.33	12.15	7.88
1, 2, 4, 5	Webcams with and without contact by palm and dorsum	29.52	30.96	19.66
1, 2, 3, 4, 5, 6	Contact and contactless by palm and dorsum sides	26.75	18.95	9.30

**Table 8.** Interoperability with two training sets and a combination at score level for finger geometry approach in terms of EER (%).

**Training Systems A**	**Training Systems B**	**Verification Systems C**	**Previous (A^1^/B^2^) EER (%) for C**	**EER (%)**
**Dorsum with contact**	**Palm with contact**	**Dorsum with contact**		
Visible webcam	Scanner	Webcam 850 nm	3.46/5.30	2.43
**Dorsum with contact**	**Palm with contact**	**Palm with contact**		
Visible webcam	Scanner	PRM	14.07/2.08	1.66
Webcam 850 nm	PRM	Scanner	14.66/8.78	1.72
**Dorsum with contact**	**Palm with contact**	**Palm contactless**		
Visible webcam	Scanner	Contactless 20–30 cm	18.00/8.44	1.66
**Palm with contact**	**Palm contactless**	**Palm contactless**		
Scanner	Contactless 20–30 cm	Contactless 5–10 cm	9.01/1.92	1.22
Scanner	Contactless 5–10 cm	Contactless 20–30 cm	8.44/1.98	1.81
**Dorsum with contact**	**Palm contactless**	**Various**		
Webcam 850 nm	Contactless 20–30 cm	PRM	9,65/13,61	5,43
Visible webcam	Contactless 20–30 cm	Webcam 850 nm	3,46/23,24	4,40
Visible webcam	Contactless 20–30 cm	Contactless 5–10 cm	15,86/1,92	1,46

1 EER training with system A and verifying with system C

2 EER training with system B and verifying with system C.

**Table 9.** Interoperability matrix for palm print approaches (OLOF and MSIFT) in terms of EER (%).

			**Training System**	**Test System**					
				**OLOF approach**			**MSIFT approach**		
	**Biometric Device**	**Resolution**		**3**	**4**	**5**	**3**	**4**	**5**
Palm	Scanner	509 × 701	3	0.17	9.61	19.7	0.86	38.2	36.2
	Contactless 20–30 cm	1,600 × 1,200	4	11.4	0.84	14.3	34.1	0.41	1.86
	Contactless 5–10 cm	1,600 × 1,200	5	23.2	16.5	0.87	36.9	0.95	0.13

**Table 10.** Interoperability matrix for multimodal approaches based on the fusion of Hand-Geometry and MSIFT in terms of EER (%).

	**Biometric Device**	**Resolution**	**Training System**	**Test System**		
				**3**	**4**	**5**
Palm	Scanner	509 × 701	3	0.01	11.5	12.6
	Contactless 20–30 cm	1,600 × 1,200	4	8.87	0.01	0.32
	Contactless 5–10 cm	1,600 × 1,200	5	9.13	0.02	0.01

**Table 11.** Survey about the acceptance and interoperability of the hand biometrics approaches proposed.

**Questions**	**Answers**				
	**1&2**	**3**	**4**	**5**	**6**
Which biometric system do you feel more comfortable with?	18%	45%	9%	3%	25%
Which biometric system do you consider the most hygienic?	2%	2%	74%	22%	0%
	**With Contact**			**Contactless**	
Would you prefer a contact or contactless biometric system?	54%			46%	
	Yes			No	
Do you believe the image of your hand violates/invades your privacy?	24%			76%	
